# Decreased Autophagy in Rat Heart Induced by Anti-β1-Adrenergic Receptor Autoantibodies Contributes to the Decline in Mitochondrial Membrane Potential 

**DOI:** 10.1371/journal.pone.0081296

**Published:** 2013-11-20

**Authors:** Li Wang, Keyi Lu, Haihu Hao, Xiaoyu Li, Jie Wang, Ke Wang, Jin Wang, Zi Yan, Suli Zhang, Yunhui Du, Huirong Liu

**Affiliations:** 1 Department of Physiology, Shanxi Medical University, Taiyuan, Shanxi, P. R. China; 2 Department of Nuclear Medicine, First Hospital of Shanxi Medical University, Taiyuan, Shanxi, P. R. China; 3 Department of Orthopaedics, Shanxi Dayi Hospital (Shanxi Academy of Medical Sciences), Taiyuan, Shanxi, P. R. China; 4 Department of Pathophysiology, Capital Medical University, School of Basic Medical Sciences, Beijing, P. R. China; University of Buenos Aires, Faculty of Medicine. Cardiovascular Pathophysiology Institute., Argentina

## Abstract

It has been recognized that changes in mitochondrial structure plays a key role in development of cardiac dysfunction, and autophagy has been shown to exert maintenance of mitochondrial homeostasis effects. Our previous study found that anti-β_1_-adrenergic receptor autoantibodies (β_1_-AABs) could lead to cardiac dysfunction along with abnormalities in mitochondrial structure. The present study tested the hypothesis that β_1_-AABs may induce the decline in mitochondrial membrane potential (ΔΨm) by suppression of cardiac autophagy, which contributed to cardiac dysfunction. Male adult rats were randomized to receive a vehicle or peptide corresponding to the second extracellular loop of the β_1_ adrenergic receptor (β_1_-AAB group, 0.4 μg/g every two weeks for 12 weeks) and treated with rapamycin (RAPA, an autophagy agonist) at 5 mg/kg/day for two days before detection. At the 4th week, 8th week and 12th week of active immunization, the rats were sacrificed and cardiac function and the levels of cardiac LC3 and Beclin-1 were detected. ΔΨm in cardiac myocytes was determined by myocardial radionuclide imaging technology and JC-1 staining. In the present study, β_1_-AABs caused cardiac dysfunction, reduced ΔΨm and decreased cardiac autophagy. Treatment with RAPA markedly attenuated β_1_-AABs-induced cardiac injury evidenced by recovered ΔΨm. Taken together, these results suggested that β_1_-AABs exerted significant decreased ΔΨm, which may contribute to cardiac dysfunction, most likely by decreasing cardiac autophagy in vivo. Moreover, myocardial radionuclide imaging technology may be needed to assess the risk in developing cardiac dysfunction for the people who have β_1_-AABs in their blood.

## Introduction

Cardiac dysfunction is the end-stage syndrome of various cardiovascular diseases. In recent years, the prevalence of cardiovascular diseases in China has increased from 31.4% in 1993 to 50.0% in 2003 [1]. Cardiovascular diseases have become the leading causes of death among Chinese adults [2] and myocardial dysfunction accounted for roughly 40% of all cardiovascular disease mortality [3]. Cardiovascular diseases, especially cardiac function deterioration, have become the main factors affecting human health in China. Despite advances in cardiac dysfunction treatment, mortality remains high, with 1-year mortality rates of nearly 38% in China [4]. There are still many unknown factors involved in the development of depressed cardiac function. Therefore, further exploration of its etiology is the key to reducing the mortality of cardiac dysfunction.

Accumulating evidence indicates that the changes in cardiac energy metabolism play an important role in the pathogenesis of cardiac function deterioration [5]. The regulation of cardiac energy metabolism is expected to become a new strategy for treatment of cardiac dysfunction [6]. As the energy source of myocardial cells, mitochondria structure and function play a central role in the maintenance of energy metabolism. Studies have confirmed that mitochondrial ATP production decreases in the failing heart and is linked with mitochondrial structural abnormalities [7] and a reduction in mitochondrial respiration [8]. Such mitochondrial dysfunction is always accompanied by alterations in cardiac cellular homeostasis and may lead to various heart diseases [9]. The mitochondrial membrane potential (ΔΨm) is currently considered an important parameter of mitochondrial function [10]. If ΔΨm is improved and energy supply is increased, it is possible that heart function may be improved. However, the cause of cardiac mitochondrial dysfunction is still elusive and thus some of the strategies to improve mitochondrial dysfunction have still not achieved the desired results [11].

In recent years, anti-β_1_-adrenergic receptor autoantibodies (β_1_-AABs) have been shown to be prevalently distributed in the sera of patients with dilated cardiomyopathy (DCM) [12], chronic Chagas heart disease [13] and heart failure caused by ischemic cardiomyopathy [14]. Binding of the speciﬁc autoantibody with the second extracellular loop of β_1_-adrenergic receptor (β_1_-AR-EC_II_) resulted in agonist-like effects, such as increasing the beating frequency of neonatal rat cardiomyocytes [15], enhancing atrial contractility [16], augmenting intracellular cyclic adenosine monophosphate (cAMP) accumulation [17] and activating the Ca^2+^ channels [18]. Ours [19] and others [12] researches have demonstrated that the long-term presence of β_1_-AABs resulted in myocardial remodeling in rats as well as impaired cardiac function. Moreover, the morphologic hallmarks of mitochondrial dysfunction that include mitochondria swelling and condensation were revealed by electron microscopy with the existence of β_1_-AABs [19]. It was not clear, however, whether the changes in ΔΨm (an indicator of mitochondrial function) were caused by β_1_-AABs. Yet another study has shown that the β-adrenergic receptor agonist isoproterenol can reduce autophagy, which is required for the maintenance of cellular homeostasis [20]. However, whether autophagy is changed with the long-term existence of β_1_-AABs remains largely unknown. If it does, what is the contribution of autophagy to the changes in ΔΨm? 

Therefore, the aims of the present study were to identify the effects of β_1_-AABs on ΔΨm and to determine whether autophagy was involved in ΔΨm changes.

## Materials and Methods

### Ethics Statement

All the animal experiments were performed in accordance with the Guide for the Care and Use of Laboratory Animals published by the US National Institutes of Health (NIH publication No. 85-23, revised 1996), approved by the Institutional Animal Care and Use Committee of Shanxi Medical University, and the Guide for the Care and Use of Laboratory Animals according to the regulation in the People's Republic of China. The Wistar rats used in the present study were obtained from the Animal Center of Shanxi Medical University, P.R.China.

### β_1_-AAB-positive rat model and rapamycin (RAPA) treatment

Animals were randomly assigned to two experimental groups: Vehicle group and β1-AAB group. A synthetic peptide of (197~223, H-W-W-R-A-E-S-D-E-A-R-R-C-Y-N-D-P-K-C-C-D-F-V-T-N-R-A, rat homology 100%, synthesized by GL Biochemical Co., LTD, Shanghai, purity was greater than 95%) was dissolved in Na_2_CO_3_ solution (concentration of 1 mg/ml), and emulsified with the same volume of complete Freund's adjuvant (CFA) (Sigma, F5881). Then, the rats in the β_1_-AAB group were injected with this antigen emulsified in CFA (0.4 μg/g, subcutaneous injection) posteriorly along the back at multiple sites for the first time. Afterwards, a booster immunization was given posteriorly along the back every two weeks with a mixture of equal volume of peptide solution and incomplete Freund's adjuvant (Sigma, F5506). The rats in the Vehicle group were injected with 1 ml of normal saline (1 mg/ml) mixed with 1 ml of incomplete Freund's adjuvant in the same manner as described above. Blood samples were collected 1 day before booster injection to test the generation of β_1_-AABs after immunization. A subset of β1-AAB animals were treated with RAPA for two days at the end of the study (β1-AAB+RAPA group). RAPA (Sigma, R0395) was dissolved in dimethyl sulfoxide (DMSO) (25 mg/ml) and further diluted with phosphate-buffered saline (PBS) before intraperitoneally (i.p) injection. The final dose was 5 mg/kg/day of RAPA.

### Enzyme-linked immunosorbent assay (ELISA) and preparation of IgG (immunoglobulin G)

Peptides corresponding to the sequence of the second extracellular loops of human β_1_-adrenoceptors were synthesized, and ELISA was performed as previously described [21]. In brief, fifty microliters of the peptide (50 µg/ml) in 100 mM Na_2_CO_3_ solution (pH 11.0) was coated on NUNC (Kastrup, Denmark) microtitre plates overnight at 4 °C. The wells were then saturated with PMT [PBS supplemented with 3% skimmed milk (W/V), 0.1% Tween 20 (V/V) and 0.01% thimerosal (W/V)] for 1 h at 37 °C. After washing the wells three times with PMT, 5 μl sera were added to 95 μl PMT solution and was incubated for 1 h at 37 °C. After washing again three times with PMT, an affinity-purified biotinylated sheep anti-rat IgG (H+L) antibody (1:2000 dilution, Beijing Zhongshan Golden Bridge Biotechnology, ZB-2040) was added and allowed to react for 1 h at 37 °C. After three times washing, the bound biotinylated antibody was detected by incubating the plates for 1 h with horseradish peroxidase streptavidin (1:3000 dilution, Vector, SA-5004). Then the plate was washed with PBS three times and the substrate [2.5 mM H_2_O_2_, 2mM 2, 2'-azinodi (ethylbenzthiazoline) sulfuric acid (ABTS, Bio Basic Inc., AD0002)] was added. Optical density was read after 30 min at 405 nm by using a microplate reader (Molecular Devices Corp., USA). The positive reaction of the sera against the peptides was confirmed as reported by Liu et al [22]. Immunoglobulin G (IgG) was affinity purified from β1-AAB-positive serum by MAbTrap™ Kit (GE Healthcare, 17-1128-01) and the total puriﬁed IgG concentration (mg/ml) was determined by the Bicinchoninic Acid (BCA) Protein Assay (thermo scientific, #23225).

### Measurement of cardiac function in vivo

The left ventricular function of rats in the β_1_-AAB group and the Vehicle group were determined at the 4th, 8th and 12th week after immunization as described previously [22]. Brieﬂy, after anesthesia, a cannula was inserted into the left ventricle via the right carotid artery to measure the following primary and derived variables, including the left ventricular systolic pressure (LVSP), left ventricular end diastolic pressure (LVEDP), and maximal positive and negative values of the instantaneous ﬁrst derivative of left ventricular pressure (+dP/dt_max_ and −dP/dt_max_).

### Myocardial radionuclide imaging

Myocardial radionuclide imaging is a technique used to reflect the ΔΨm [23]. Myocardial uptake of ^99m^Tc-methoxyisobutylisonitrile (^99m^Tc-MIBI) was measured as H/M count ratio, by use of regions of interest positioned over the heart (H) and upper mediastinum (M) [24]. The decline in cardiac ^99m^Tc-MIBI uptake means decreased ΔΨm. 

A 18.5-MBq dose of ^99m^Tc-MIBI was injected slowly through the rat tail vein. The planar and single photon emission computed tomography views were obtained approximately 30 min after injection. For image processing, we used a conventional gamma scintillation camera (Mobile Radioisotope Camera, Model BHP6602, Hamamatsu, Japan), equipped with a low-energy, high-resolution collimator and selected a 20% energy window encompassing the 140 keV photopeak. Imaging was performed by projection reconstruction with the chest open and collected in a 256 × 256 matrix format. 

### Myocyte isolation and culture

At the 4th week after immunization, rats from the Vehicle group, β1-AAB group and β1-AAB+RAPA group were heparinized (1000 U/ kg) and anaesthetized. The hearts were excised and the aorta was cannulated and immediately perfused on a Langendorff apparatus. Hearts were initially perfused for a 5 min period with Ca^2+^-free Tyrode's solution, followed by a 25 min period with Ca^2+^-free Tyrode’s solution containing collagenase B and collagenase D (Roche Chemical Co.). The ventricles were then excised, rinsed and cut into small pieces in Ca^2+^-free Tyrode's solution supplemented with 0.1mM CaCl_2_ and 1% BSA). The cells were collected using 50 mesh screen filter and the supernatant was centrifuged at 50g for 1min at room temperature and then resuspended for a three-step Ca^2+^ restoration procedure (i.e. 0.1mM, 0.5mM, 1mM). The freshly isolated cardiomyocytes were then suspended in M199 medium (Hyclone, SH30253.01B) and incubated at 37 °C in a 5% CO_2_ atmosphere. 

### Cell culture and RAPA treatment

Rat cardiomyocyte-derived cell line H9c2 was purchased from Cell Bank of China Science Academy (Shanghai, China). Cells were cultured in Dulbecco's modified Eagle's medium (DMEM) (Hyclone, SH30022.01B) containing 10% fetal bovine serum (FBS) (Sijiqing, W0001), 100 U/mL penicillin and 100 μg/mL streptomycin (Solarbio, P1400-100) and incubated at 37 °C in a 5% CO_2_ atmosphere. H9c2 Cells were treated with β1-AABs (1μmol/L) for 24 hours or pretreated with 10 ng/mL RAPA for 30 minutes and then treated with β1-AABs (1μmol/L) for 24 hours in the continued presence of RAPA. The vehicle to administer RAPA was DMSO (0.1% total volume).

### JC-1 staining

JC-1 (Beyotime Biotech, C2006), a sensitive fluorescent probe for ΔΨm, was employed to measure the ΔΨm of cardiomyocytes. In healthy cells with high mitochondrial ΔΨm, JC-1 spontaneously forms complexes known as JC-1 aggregates with intense red fluorescence. On the other hand, in apoptotic or unhealthy cells with low ΔΨm, JC-1 remains in the monomeric form, which shows only green fluorescence. According to the manufacturer's directions, after indicated treatments, cells were incubated with JC-1 staining solution (5 μg/mL) for 20min at 37 °C and then rinsed twice with JC-1 staining buffer. The images were viewed and scanned under laser confocal microscopy (OLYMPUS, FV1000, Japan) at 488 nm excitation and 530 nm emission for green, and at 543 nm excitation and 590 nm emission for red. All the parameters used in confocal microscopy were kept constant in each sample, such as laser power, pinhole, gain, scan speed and offset. Four repeated measurements were performed. In addition, to quantify JC-1 data, 100 μl of cell sample was used to read the fluorescence in 96-well clear-bottom black plate (Corning, #3603) at Ex488/Em530 and Ex543/Em590 as previously described [25] in SpectraMax® M2e Microplate Reader (Molecular Devices, Sunnyvale, CA, USA). The ratio of red to green fluorescence was calculated as ΔΨm. Mitochondrial depolarization is indicated by an decrease in the red/green ﬂuorescence intensity ratio. In addition, the percentage of low Δψm H9c2 cells were assessed by ﬂow cytometry (BD FACSCanto, NJ, USA). The X-axis (FL-1 channel) of flow cytometry results indicated the green fluorescence intensity (JC-1 monomers) and the Y-axis (FL-2 channel) was used to detect the red fluorescence (JC-1 aggregates). The R2 (region 2) encloses the low ΔΨm cell population.

### Western blot

The protein levels of Beclin-1 and LC3 were detected by western blot. For western immunoblotting studies, cardiac tissue (70 mg) was lysed in RIPA buffer (Beyotime Biotech, P0013B). Protein concentration was determined by BCA Protein Assay Kit (Thermo scientific, 23225) and 40 μg of protein was resolved on a 12% SDS-PAGE gel, electrophoresed (80 V for 30 min followed by 120 V for 90 min), and transferred to a polyvinylidene difluoride (PVDF) membrane (Millipore, IPVH00010). The membrane was blocked for 2 h at room temperature in Tris-buffered saline (TBS) pH 7.4/Tween 0.1% containing 0.3% gelatin (Sigma-aldrich, A9418), and then incubated using the primary antibodies at 4°C overnight followed by detection with the second antibodies. The following primary antibodies were used: anti-Beclin-1 monoclonal antibody (dilution 1:1000, Santa Cruz, sc-48341) or anti-LC3B polyclonal antibody (dilution 1:1000, Cell Signaling, 2775) or anti-β-actin monoclonal antibody (dilution 1:1000, Sigma-aldrich, A1978). Binding of specific antibody was detected with a horseradish peroxidase-conjugated anti-mouse IgG or peroxidase-conjugated anti-rabbit IgG at a dilution of 1:3000 for 1 h (Beijing Zhongshan Golden Bridge Biotechnology, ZB-2305, ZB-2301). Specific antibody binding was detected using electrochemiluminescence. The density of the scanned protein bands was measured by image analysis software and the results were presented as a percentage change of the loading control. 

### Real-time PCR

mRNA expressions of Beclin-1 and LC3 were detected by Real-time PCR with SYBR Premix Ex TaqTM II (TaKaRa, DRR820A) detection in the Stratagene MX 3005P real-time PCR system. Total RNA was isolated from the left ventricle for analysis using the RNAiso plus (TaKaRa, D9108B). 3μg of total RNA was reversely transcribed into cDNA. The thermal profile for SYBR Green PCR was 95°C for 30s, followed by 40 cycles of denaturation at 95°C for 5s and annealing/extension at 60°C for 20s. The primer sequences were as follows: LC3, sense: 5’-CATGCCGTCCGAG-AAGACCT-3’ and antisense: 5’-GATGAGCCGGACATCTTCCACT-3’ (GenBank TM accession number, NM022867.2); Beclin-1, sense: 5’-TTGGCCAATAAGATGG-GTCTGAA-3’ and antisense: 5’-TGTCAGGGACTCCAGATACGAGTG-3’ (GenBankTM accession number, NM001034117.1). Samples were normalized against GAPDH expression to ensure equal loading. The specificity of the amplified product was monitored by its dissociation curve. The results, expressed as the fold difference in the number of LC3 or Beclin-1 copies relative to the number of GAPDH gene copies, were determined by the relative quantitative 2^-ΔΔCt^ method. ΔΔCt = ΔCt (target gene)-ΔCt (GAPDH) and ΔCt (target gene) = Ct (experimental-target)-Ct (control-target) and ΔCt (GAPDH) = Ct (experimental-GAPDH)-Ct (control- GAPDH).

### Immunofluorescence

Beclin-1 and LC3 were detected by immunofluorescence. Myocardial tissue samples were embedded in Tissue-Tek OCT compound (Sakura Finetechnical Co., 4583) and were sectioned at 10 μm thickness with cryostat (CM3050 S, Leica, Deerﬁeld, IL), air-dried for 60 min, fixed with acetone for 15 min at 4°C and stored at -20°C until used. These frozen sections were incubated with antibodies against LC3B (1:400; Cell Signaling, 2775) and Beclin-1 (1:50, Santa Cruz Biotechnology, sc-48341) in a humidified container at 4°C overnight. After washing with PBS, the frozen sections were incubated with tetramethylrhodamine isothiocyanate (TRITC)-conjugated second antibodies. TRITC-labeled second anti-rabbit IgG (1:50) and TRITC-labeled second anti-mouse IgG (1:50) were from Beijing Zhongshan Golden Bridge Biotechnology (ZF-0316, ZF-0313). After washing three times with PBS, 2-(4-Amidino-phenyl)-6-Indolecarba-midine dihydrochloride (DAPI, Beyotime Biotech, C1005) solution was added to stain the cell nucleus for 3 min. Sections were then washed in PBS and sealed with a coverslip. The slides were analyzed with laser confocal microscopy (OLYMPUS, FV1000, USA). 

### Statistical analysis

Data were presented as mean ± standard deviation (SD). Statistical analysis was performed with the SPSS 15.0 program. *T*-tests were used to compare two independent sample means, and one-way ANOVA with Bonferroni *post hoc* tests were performed for comparing means of more than two samples. Statistical significance was defined as p < 0.05.

## Results

### β_1_-AAB-positive rat models were established

**Figure 1:**
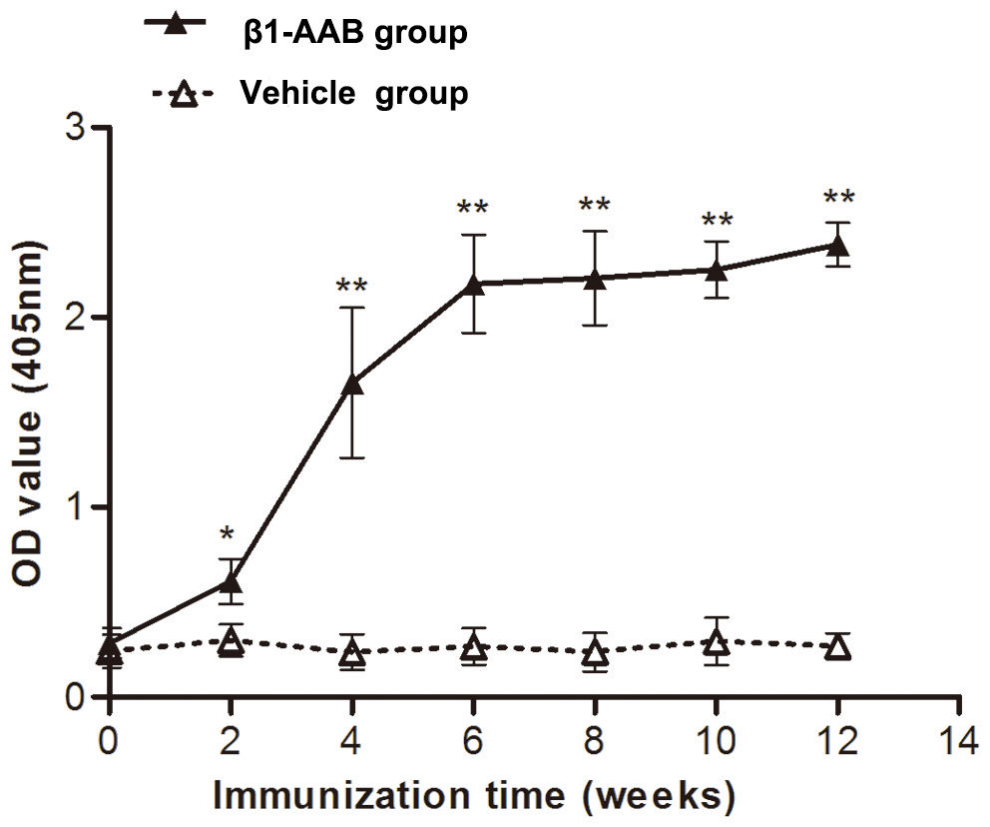
The generation of β_1_-AABs after active immunization against β_1_-AR-EC_II_. Adult rats were actively immunized with β_1_-AR-EC_II_. Antibody titer is defined by OD value. Data are expressed as Mean ± SD (n=12 per group). *P < 0.05; **P < 0.01.

In both experimental groups, the OD value of β_1_-AABs in the sera before treatment were at a very low level. However, these were markedly increased in the β_1_-AAB group after two weeks of immunization. Moreover, the serum levels remained relatively high until the end of the experiment (Figure 1).

### Cardiac function was decreased with the existence of β_1_-AABs

**Figure 2:**
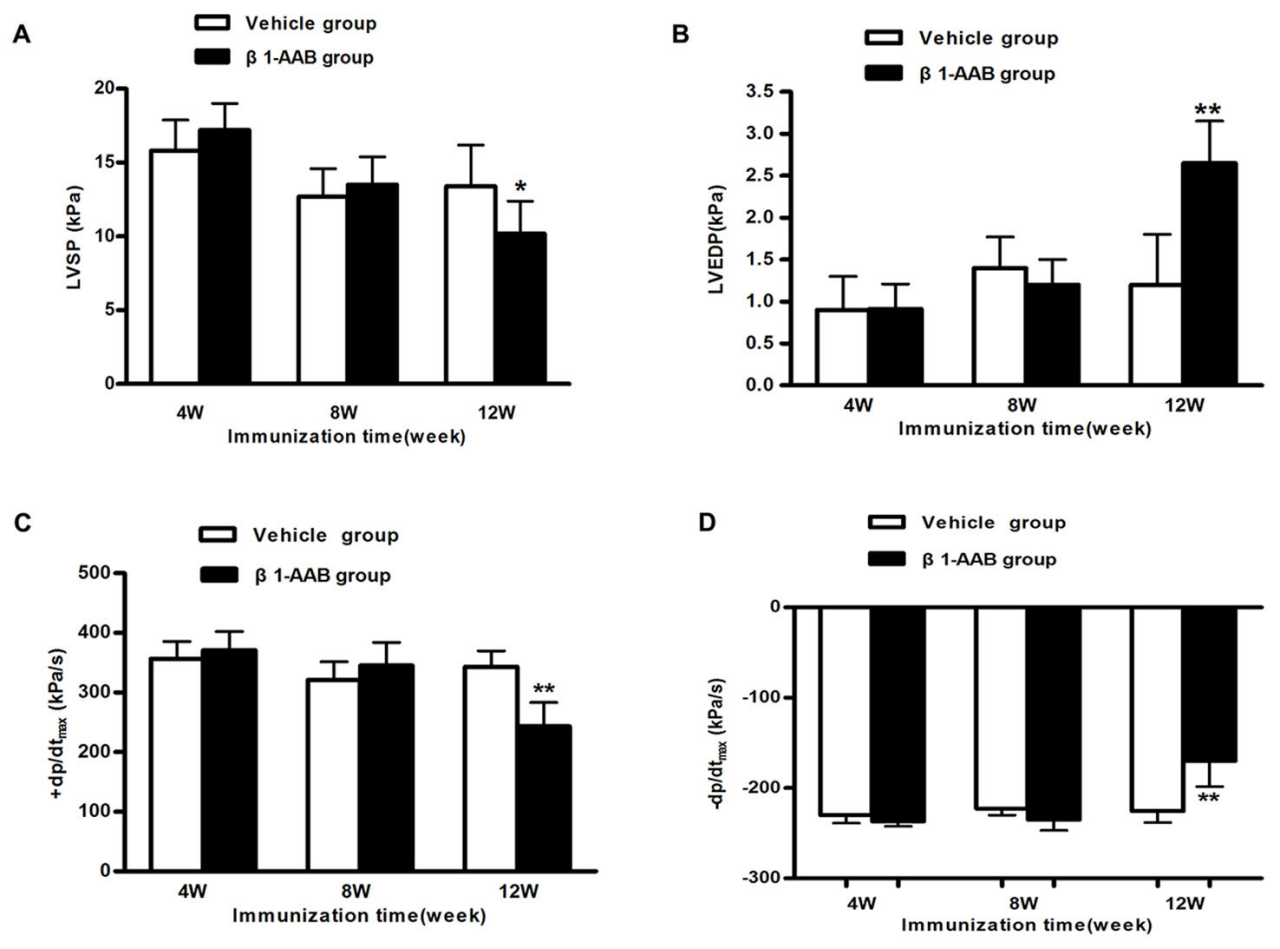
Assessment of cardiac function at different time points (4w, 8w and 12w) after active immunization. Comparison of LVSP (A), LVEDP (B), +dp/dt_max_ (C) and −dp/dt_max_ (D) between the rats in the Vehicle group and β_1_-AAB group. Data are expressed as Mean ± SD (n=8 per group). *P < 0.05; **P < 0.01.

At the 4th week and 8th week, no obvious change has been found on the left ventricular function parameters. However, at the 12th week after immunization, the left ventricular systolic and diastolic functions, expressed by LVSP, +dP/dt_max_ and LVEDP, -dP/dt_max_, decreased signiﬁcantly in the β1-AAB group compared with the Vehicle group (Figure 2A-D). These results indicated that cardiac function was declined as a result of immunization.

### The ΔΨm in rat cardiac myocytes declined with the existence of β_1_-AABs

The ΔΨm is an important parameter of mitochondrial function [10]. In this study, myocardial radionuclide imaging technology and JC-1 staining were executed to evaluate the alteration in ΔΨm.

**Figure 3:**
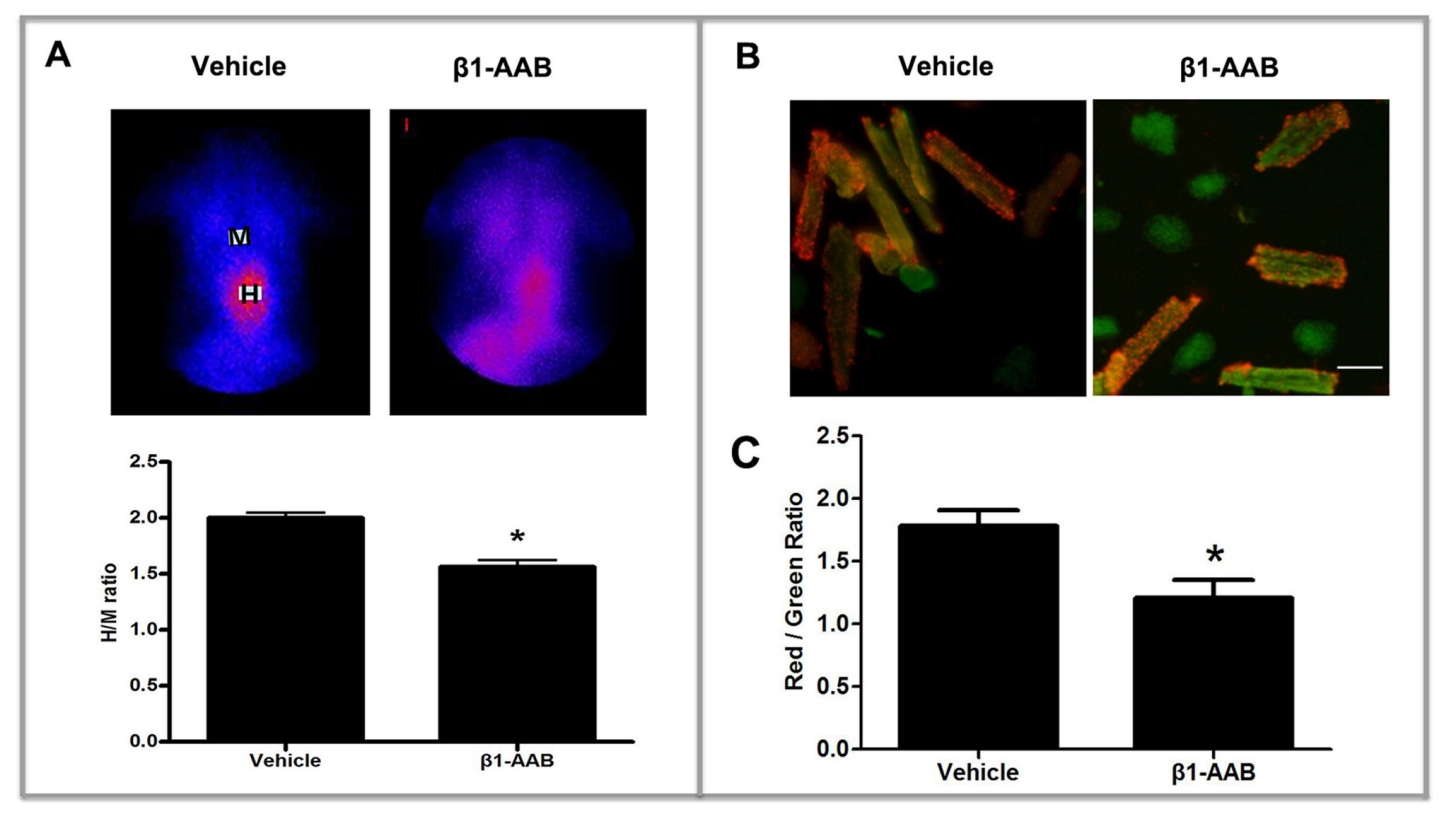
Assessment of ΔΨm at the 4th week after active immunization. (A) Representative images of cardiac radionuclide imaging. Cardiac ^99m^Tc-MIBI uptake was measured as H/M count ratio, by use of regions of interest positioned over the heart (H) and upper mediastinum (M). Data are expressed as Mean ± SD (n=6 per group). *P < 0.05. (B) Representative images of JC-1 staining. The adult rat ventricular cardiomyocytes were isolated, stained with JC-1 and then the stained cells were imaged using confocal microscopy. Scale bar: B = 50 μm. (C) Quantitative analysis of the shift of mitochondrial red ﬂuorescence to green ﬂuorescence among groups. The fluorescence was measured using a SpectraMax M2e microplate reader and the ratio of red to green fluorescence was calculated as ΔΨm. Data are expressed as Mean ± SD (n=6 per group). *P < 0.05.

**Figure 4:**
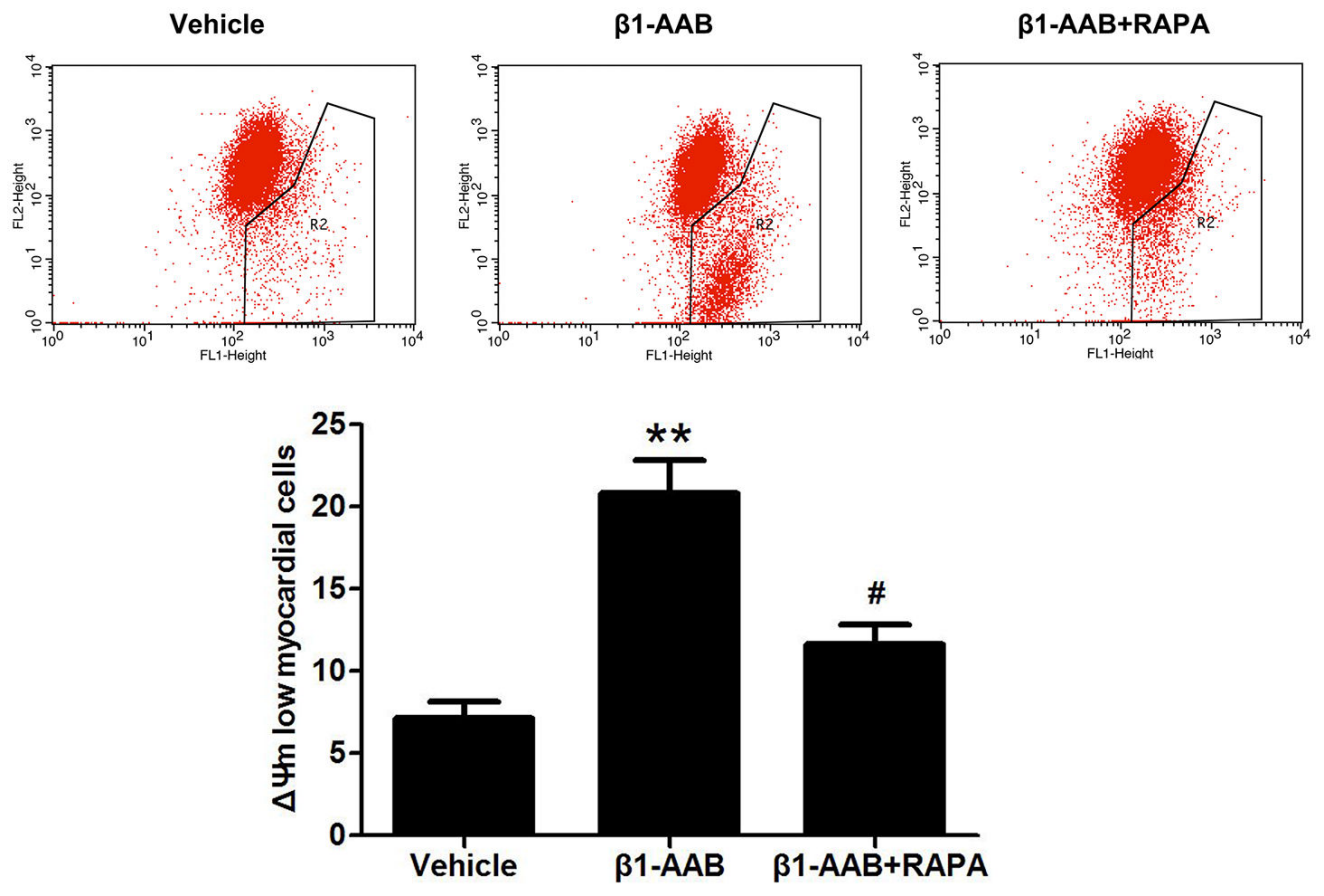
Changes in ΔΨm of cardiomyocytes (H9c2 Cells) with the existence of β_1_-AABs and RAPA. Representative images of JC-1 ﬂuorescence with ﬂow cytometry and quantitative analysis of ΔΨm. After β_1_-AAB treatment, decreased ΔΨm was indicated by increased cell population in R2. Induction of autophagy by RAPA lead to the restoration of red ﬂuorescence indicated by decreased cell population in R2. Data are expressed as Mean ± SD (n=6 per group). **P < 0.01 *vs*. Vehicle; ^#^P < 0.05 *vs*. β_1_-AAB group.

Myocardium uptake of ^99m^Tc-MIBI, expressed by the H/M ratio [regions of interest positioned over the heart (H) and upper mediastinum (M)] [24], can be used to reflect the ΔΨm in cardiac myocytes. As shown in Figure 3A, the H/M ratio declined at the 4th week after immunization and this trend continued until the end of the study in β_1_-AAB group versus the Vehicle group (Results are not reported). Meanwhile, JC-1 staining was used to detect the ΔΨm. When the ΔΨm is high, JC-1 accumulates in the mitochondrial matrix to form JC-1 aggregates that produce red ﬂuorescence. Alternatively, green ﬂuorescence is generated by the JC-1 monomers when JC-1 cannot assemble in the mitochondrial matrix. In the present study, at the 4th week after immunization, ventricular cardiomyocytes were isolated and stained with JC-1. After adding JC-1, the intensity of the red fluorescence of JC-1 aggregates was signiﬁcantly weaker and the green fluorescence of JC-1 monomers was stronger in the β_1_-AAB group compared with the Vehicle group (Figure 3B, C). To further confirm these results, H9c2 cells, derived from embryonic rat heart, were treated with β1-AABs (1μmol/L) for 24 hours and stained with JC-1, followed by flow cytometry. The data showed that the ΔΨm rapidly decreased, as shown by increased cell population in R2 (Figure 4). These results demonstrated that the ΔΨm was declined with the existence of β_1_-AABs.

### Decreased myocardial autophagy contributes to the decline in ΔΨm with the existence of β_1_-AABs

**Figure 5:**
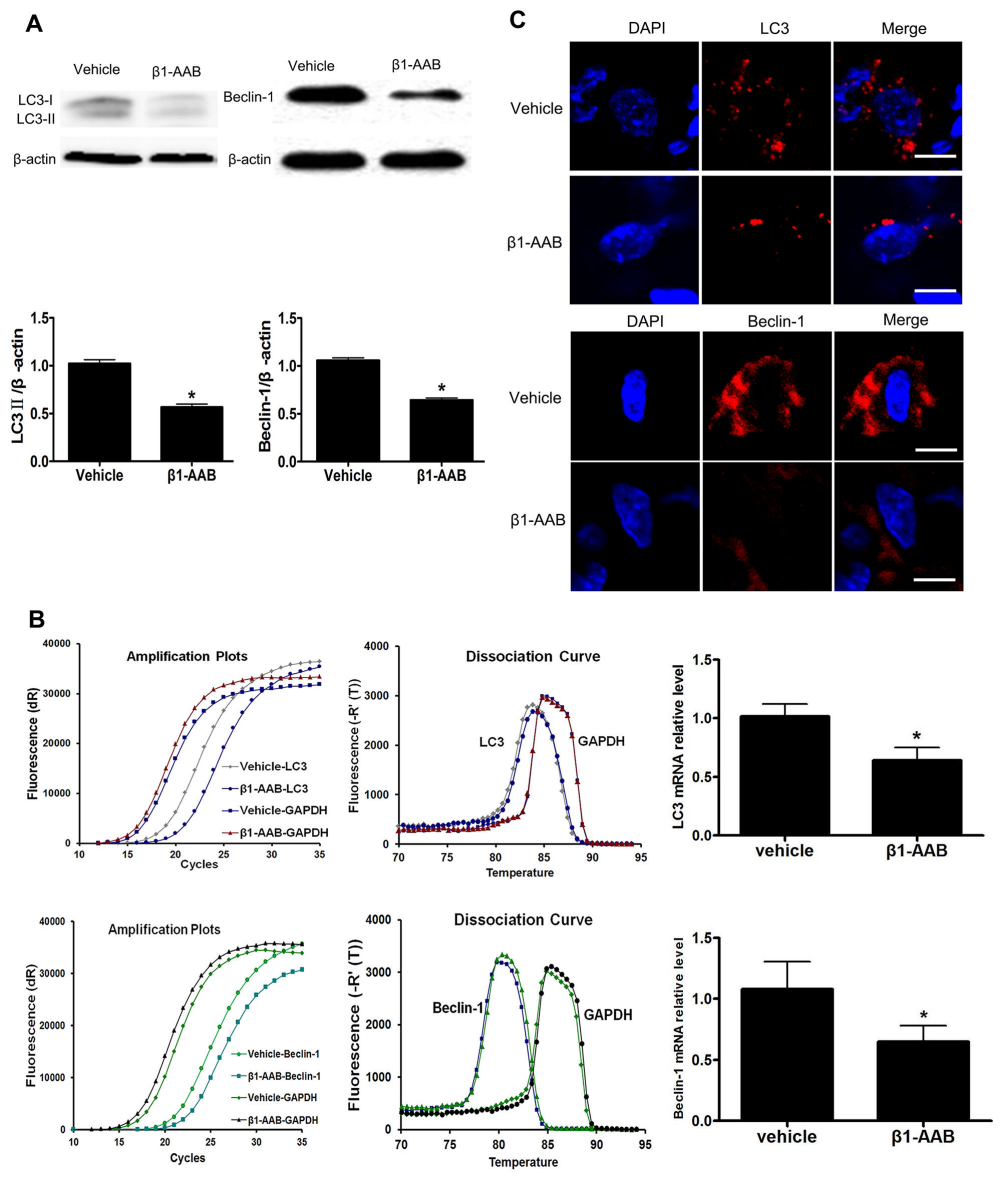
The level of autophagy decreased with the existence of β_1_-AABs at the 4th week. (A and B) The levels of LC3 and Beclin-1 protein and mRNA expression. Data are expressed as Mean ± SD (n=6 per group). *P < 0.05. (C) Beclin-1 and LC3 immunoreactivity were present as punctate pot (red) and the nucleus were stained with DAPI (blue). Scale bar: C=10 μm.

**Figure 6:**
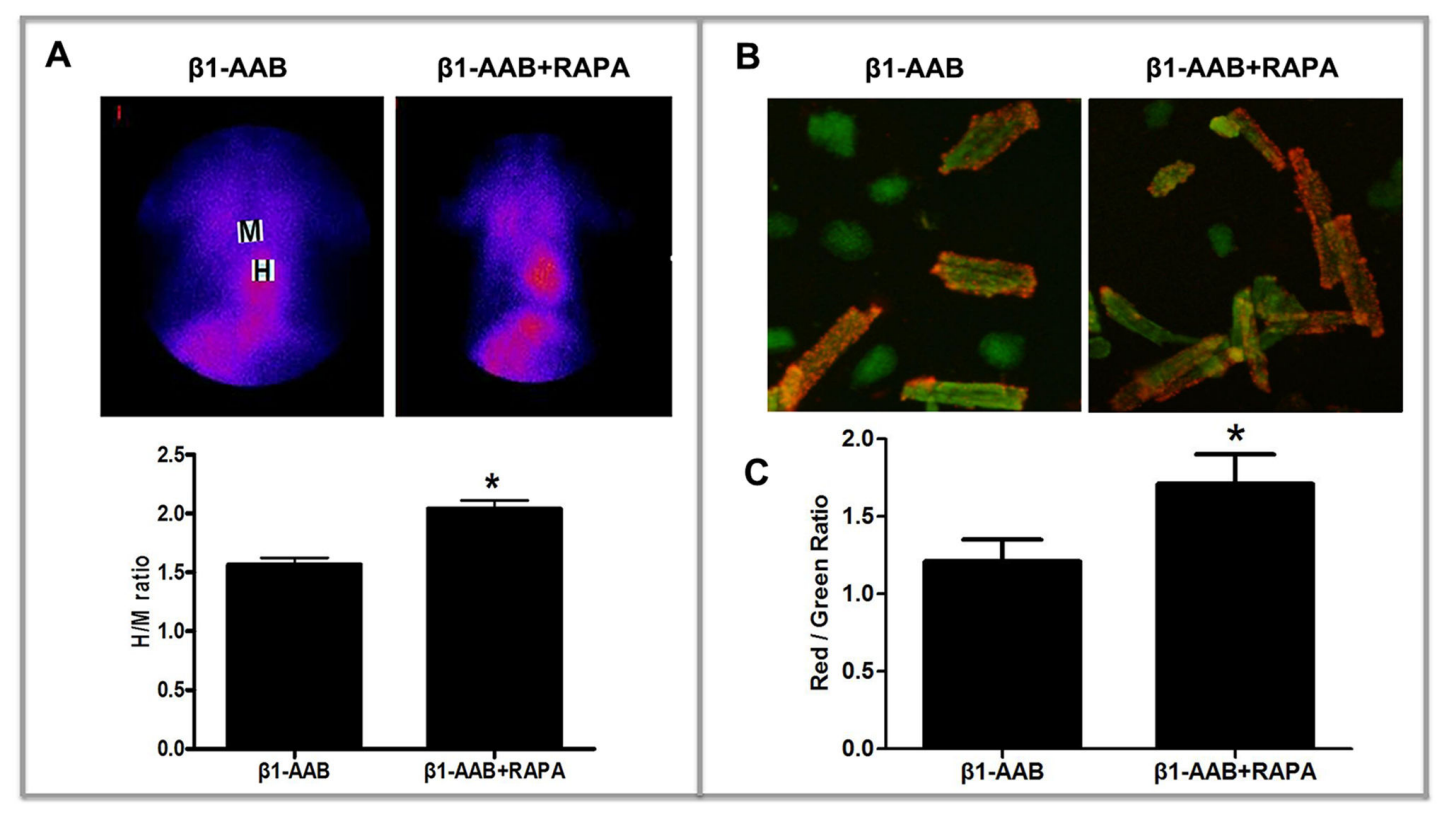
Induction of autophagy by RAPA can improve the ΔΨm in cardiomyocytes. (A) Induction of autophagy by RAPA results in enhanced myocardium uptake of ^99m^Tc-MIBI. Data are expressed as Mean ± SD (n=6 per group). *P < 0.05. (B) Induction of autophagy can increase red fluorescence intensity of JC-1 aggregates. Scale bar: B=50 μm. (C) Induction of autophagy increase the ratio of red to green fluorescence. Data are expressed as Mean ± SD (n=6 per group). *P < 0.05.

To determine the extent of autophagy are influenced by β_1_-AABs, LC3 and Beclin-1, which have been used as molecular markers of autophagic activity, were measured in the present study. Both protein (Figure 5A) and mRNA levels (Figure 5B) of LC3 and Beclin-1 were significantly decreased at the 4th week in the β_1_-AAB group compared with the Vehicle group. Moreover, compared with the Vehicle group, the Beclin-1 and LC3 punctate dots were significantly reduced in the β1-AAB group by immunofluorescence staining (Figure 5C). Meanwhile, β1-AR-ECII treatment did not affect the level of autophagy and cell survival in H9c2 cardiac cells (File S1, Figure S1 and Figure S2). To further evaluate the role of decreased autophagy in ΔΨm, RAPA, an mTOR inhibitor, was used to enhance autophagy [26]. β_1_-AAB animals were treated with RAPA (β1-AAB+RAPA group) at 5 mg/kg/day for two days before detection. As shown in Figure 6A, pretreatment with RAPA significantly increased the myocardium uptake of ^99m^Tc-MIBI. Meanwhile, cultured cardiac myocytes were isolated from the rats in β1-AAB+RAPA group and stained with JC-1 showing the restoration of ΔΨm, as indicated by the increase of the red : green fluorescence ratio (Figure 6B, C). To further confirm the result, cardiomyocytes (H9c2 Cells) were pretreated with 10 ng/mL RAPA or DMSO vehicle (0.1% total volume) for 30 minutes and then treated with β1-AABs (1μmol/L) for 24 hours in the continued presence of RAPA or DMSO. As shown in Figures S3 (A-C) and S4, pretreatment with RAPA increased the level of autophagy whereas DMSO vehicle alone did not affect the level of autophagy in β1-AAB-treated H9c2 cardiomyocytes. In addition, β1-AAB+RAPA-treated H9c2 cells were stained with JC-1 (Figure 4). The data showed an increase in ΔΨm as indicated by decreased cell population in R2. Taken together, these results demonstrated that the induction of autophagy could improve ΔΨm in cardiac myocytes with declined ΔΨm due to the existence of β1-AABs.

## Discussion

In the present study, we demonstrated that the long-term existence of β_1_-AABs in rat resulted in decreased autophagy and the decline in ΔΨm that was earlier than the emergence of abnormal cardiac function. Additionally, activation of autophagy by RAPA can effectively improve the ΔΨm in cardiac myocytes.

Since the 1990s, β_1_-AABs were frequently detected in patients suffering from dilated cardiomyopathy (DCM) [17] and Chagas' heart disease [27]. In a previous study, we detected these autoantibodies in a rat model of heart failure [22] and found that they could influence heart function [19]. In a previous paper [19], when the immunization time was once a month, we demonstrated that the left ventricular systolic and diastolic functions decreased significantly in β1-AAB group at the 18th month after immunization. In the current study, the immunization time we chose is once every two weeks. Because of such a high frequency of immunization, the OD value of β_1_-AABs in the sera were markedly increased and the heart function aggravated progressively in the β_1_-AAB group at the 12th week after immunization. 

+dp/dt_max,_ which was used as an index of myocardial contractility, started to decrease at 12 weeks after immunization showing that the existence of β_1_-AABs could reduce cardiac contractility. Among many factors affecting myocardial contractility, mitochondria regulate cardiac cell contractility by providing ATP for cellular ATPases and by participating in Ca^2+^ homeostasis [28]. Mitochondria dysfunction results in bioenergetic defect and cardiac function deterioration. 

The ΔΨm, which is the electrochemical gradient that is present across the inner mitochondrial membrane, is critical for maintaining the physiological function of the respiratory chain to generate ATP [29], so the ΔΨm reflects the integrity of mitochondrial function and is a key indicator of mitochondrial function [10]. In the present study, the ΔΨm was measured through use of the cardiac radionuclide imaging and the lipophilic mitochondrial probe JC-1.

Cardiac radionuclide imaging uses an intravenous radiopharmaceutical to generate scintigraphic images of the myocardium and the images are acquired using a gamma camera. ^99m^Tc-MIBI, which is a lipophilic cationic myocardial perfusion imaging agent, can move across the cytoplasmic membrane and the mitochondrial membrane by passive diffusion in response to transmembrane potential. Approximately 90% of myocardial ^99m^Tc-MIBI is localized within the mitochondrial fraction [30]. Most of the accumulated ^99m^Tc-MIBI is related to mitochondrial uptake. Myocardial uptake of ^99m^Tc-MIBI is dependent on ΔΨm . When the ΔΨm is depolarized, the myocardial uptake of ^99m^Tc-MIBI will be inhibited [23], so the myocardial uptake of ^99m^Tc-MIBI is used to reflect the ΔΨm and mitochondrial function. The advantage of ^99m^Tc-MIBI scanning is that it is a noninvasive inspection and can be better used in clinical practice. In the present study, at the 4th week after immunization, the myocardium uptake of ^99m^Tc-MIBI declined significantly. The existence of β_1_-AABs caused the disruption of the ΔΨm and may contribute to cardiac dysfunction. Meanwhile, JC-1 staining was used to detect the ΔΨm in cardiac myocytes. JC-1 has been reported to be a more reliable indicator of ΔΨm than other dyes described herein [31,32] and can selectively enter into mitochondria and reversibly change color from red to green as the membrane potential decreases. The ratio of red to green fluorescence of JC-1 depends only on the ΔΨm [33] and can therefore be used as an indicator of ΔΨm [25,34-37]. Our results also found that ΔΨm was decreased, expressed by the increase in green ﬂuorescence and the concomitant attenuation of red ﬂuorescence in the β_1_-AAB group compared with the Vehicle group. Flow cytometric analysis of cardiomyocytes (H9c2 Cells) showed similar results. As can be seen from the above results, the loss of ΔΨm induced by β_1_-AABs that emerged in the 4th week was earlier than the declined cardiac function that started to emerge in the 12th week. It has been reported that β_1_-AABs can be detected in the serum of approximately 10% of healthy human populations [21]. If the functional state of the mitochondria can be evaluated by noninvasive radionuclide scan for those with normal heart function that detected by electrocardiogram, myocardial enzymes, echocardiography and X-ray but β_1_-AAB-positive, the potentially dangerous conditions that can cause cardiac dysfunction may be prevented earlier.

Mitochondrial dysfunction often leads to diverse cellular responses, including autophagy. The autophagic response, in return, plays a pivotal role in mitochondrial degradation [38]. Decreased ΔΨm could promote mitochondrial dysfunction [39] and induce activation of autophagy [40]. More importantly, autophagy can selectively remove damaged mitochondria to protect cells [39-41]. Impaired autophagy causes the accumulation of dysfunctional organelles such as mitochondria within the cell [42]. Overexpression of LC3, a well-known marker for autophagy, resulted in an improved ΔΨm and enhanced ATP production [43]. Autophagy, a lysosomal degradation pathway that is essential for survival, plays an important role in maintaining cellular homeostasis [44]. Microtubule-associated protein 1 light chain 3 (MAP1-LC3, LC3) is an essential component of the autophagic vacuoles, forming a reliable marker of autophagic activity [45]. There are three isoforms of LC3 in mammalian cells, LC3A, LC3B and LC3C, but only LC3B-II is associated with autophagic vesicle numbers, and therefore the anti-LC3B antibodies were recommended to use for analysis [46]. That is why anti-LC3B antibodies were used for autophagy assays in this study. Upon induction of autophagy, LC3-I, a cytosolic form of LC3, is converted to LC3-II (a lipidated form of LC3). LC3-II is localized in autophagosome membranes and the increase in LC3-II indicates the accumulation of autophagosomes, so LC3-II has been deemed a marker of autophagy [47]. As part of a class III PI3K complex [48], Beclin-1 is thought to be important in mediating the localization of other autophagy proteins to pre-autophagosomal structures [49]. Therefore, it is commonly used during autophagy detection.

Many studies have shown that the level of autophagy was altered in several cardiovascular diseases [50]. Moreover, autophagy was activated by propranolol that is a non-selective β-adrenergic receptor blocking agent [51] and was inhibited by the β-adrenergic agonist isoproterenol [20]. Thus, we speculate that the alteration of the β-adrenergic receptor may result in the change of autophagy. However, whether autophagy alteration is accompanied by ΔΨm and decreased cardiac function caused by β_1_-AABs remains unknown. If it does, we aimed to understand the contribution of autophagy to the changes in ΔΨm.

In the present study, the expression of LC3 and Beclin-1, markers of the autophagic process, declined with the existence of β_1_-AABs. Interestingly, induction of autophagy by RAPA results in the restoration of ΔΨm, expressed by the increase of myocardium uptake of ^99m^Tc-MIBI and the repression in green fluorescence accompanied by the restoration in red ﬂuorescence. Flow cytometric analysis of cardiomyocytes (H9c2 Cells) showed similar results. These results suggested that decreased autophagy was involved in the loss of ΔΨm.

In the present study, rat models with β_1_-AABs were established by active immunization which is the induction of immunity after exposure to a synthetic peptide corresponding to β_1_-AR-EC_II_. However, we cannot rule out the possibility that antigenic peptides themselves may influence the heart. Thus, the H9c2 cell line, derived from the embryonic rat heart, was treated with β_1_-AR-EC_II_ for 24 hours and the data indicated that the levels of autophagy and cell survival did not change significantly. 

We concede that there are some limitations to our study and further research is required. Firstly, we only observed the phenomenon that decreased ΔΨm was caused by β_1_-AABs. However, whether the changes in ΔΨm induced by β1-AABs were associated with mitochondrial dysfunction, mitochondrial damage or disorders of energy metabolism need to be further validated. We will do further research to confirm our results. Secondly, we found that the induction of autophagy contributed to improving the ΔΨm, but whether this restoration induced an improvement of the left ventricular systolic and diastolic functions requires further study. Even so, it can also be concluded that the decreased ΔΨm was induced by β_1_-AABs in which autophagy plays an important regulatory role. Our preliminary observations may open new insights into the pathogenesis and prevention of β_1_-AAB-positive heart dysfunction.

## Supporting Information

Figure S1 **The autophagy did not change with β_1_-AR-EC_II_ in the rat cardiomyocyte-derived cell line H9c2.** H9c2 cells were incubated with β_1_-AABs and β_1_-AR-EC_II_ for 24 hours and then lysised, and mRNA levels of LC3 (A) and Beclin-1 (B) were detected with Real-time PCR. Data are expressed as Mean ± SD (n=6 per group). *P < 0.05.
(TIF)

Figure S2 **β_1_-AR-EC_II_ did not significantly alter survival of H9c2 cells.** After being stimulated for 24 hours by β_1_-AABs, the level of H9c2 cell survival declined significantly and had no change in the absence of β_1_-AR-EC_II_. Data are expressed as Mean ± SD (n=6 per group). *P < 0.05.
(TIF)

Figure S3 **RAPA can induce autophagy in β1-AAB-treated rats.** (A and B) The differences in LC3 and Beclin-1 protein and mRNA expression after treatment with RAPA. (n=6 per group) (C) Confocal images of Beclin-1 and LC3. The red punctate pots recovered by RAPA. *P < 0.05 *vs*. Vehicle; ^#^P < 0.05 *vs*. β_1_-AAB group. Scale bar: C=10 μm.
(TIF)

Figure S4 **Pretreatment with RAPA increased the level of autophagy in β1-AAB-treated H9c2 cardiomyocytes.** The differences in LC3 and Beclin-1 protein expression after treatment with RAPA. Data are expressed as Mean ± SD (n=6 per group). *P < 0.05 *vs*. Vehicle; ^#^P < 0.05 *vs*. β_1_-AAB group.
(TIF)

File S1 **Supplemental materials and methods.**
(DOC)
