# Supplementary figures and images for: Decreased Autophagy in Rat Heart Induced by Anti-β1-Adrenergic Receptor Autoantibodies Contributes to the Decline in Mitochondrial Membrane Potential
Source: PLoS One. 2013 Nov 20;8(11):e81296. doi: 10.1371/journal.pone.0081296 (PMC3835737; doi:10.1371/journal.pone.0081296)

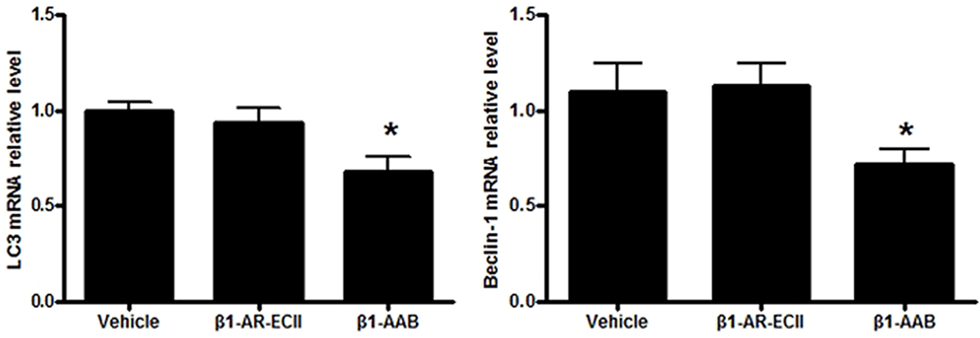

Supplement: Figure S1 — The autophagy did not change with β1-AR-ECII in the rat cardiomyocyte-derived cell line H9c2. H9c2 cells were incubated with β1-AABs and β1-AR-ECII for 24 hours and then lysised, and mRNA levels of LC3 (A) and Beclin-1 (B) were detected with Real-time PCR. Data are expressed as Mean ± SD (n=6 per group). *P < 0.05. (TIF) [file pone.0081296.s001.tif]

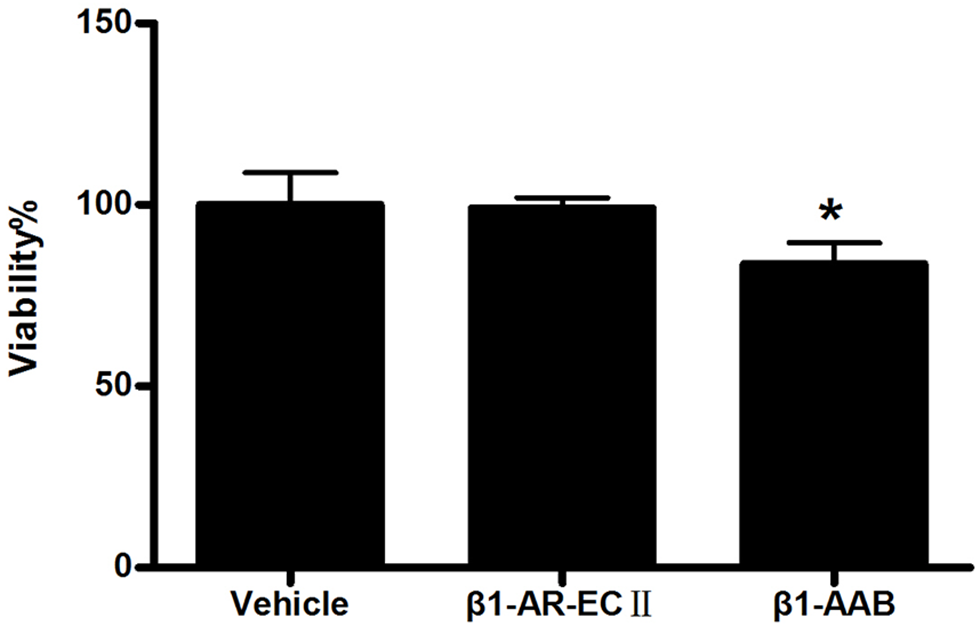

Supplement: Figure S2 — β1-AR-ECII did not significantly alter survival of H9c2 cells. After being stimulated for 24 hours by β1-AABs, the level of H9c2 cell survival declined significantly and had no change in the absence of β1-AR-ECII. Data are expressed as Mean ± SD (n=6 per group). *P < 0.05. (TIF) [file pone.0081296.s002.tif]

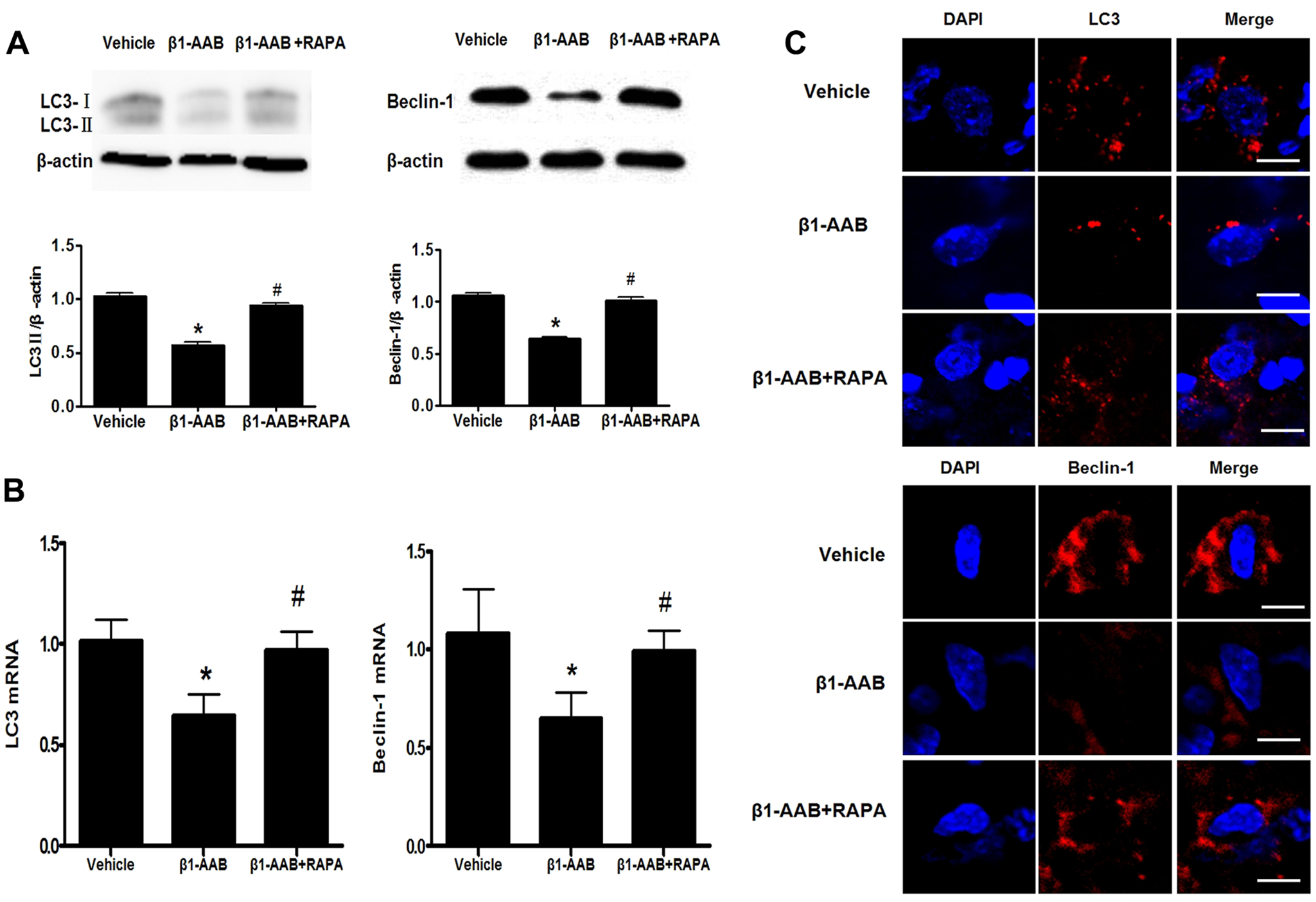

Supplement: Figure S3 — RAPA can induce autophagy in β1-AAB-treated rats. (A and B) The differences in LC3 and Beclin-1 protein and mRNA expression after treatment with RAPA. (n=6 per group) (C) Confocal images of Beclin-1 and LC3. The red punctate pots recovered by RAPA. *P < 0.05 vs. Vehicle; #P < 0.05 vs. β1-AAB group. Scale bar: C=10 μm. (TIF) [file pone.0081296.s003.tif]

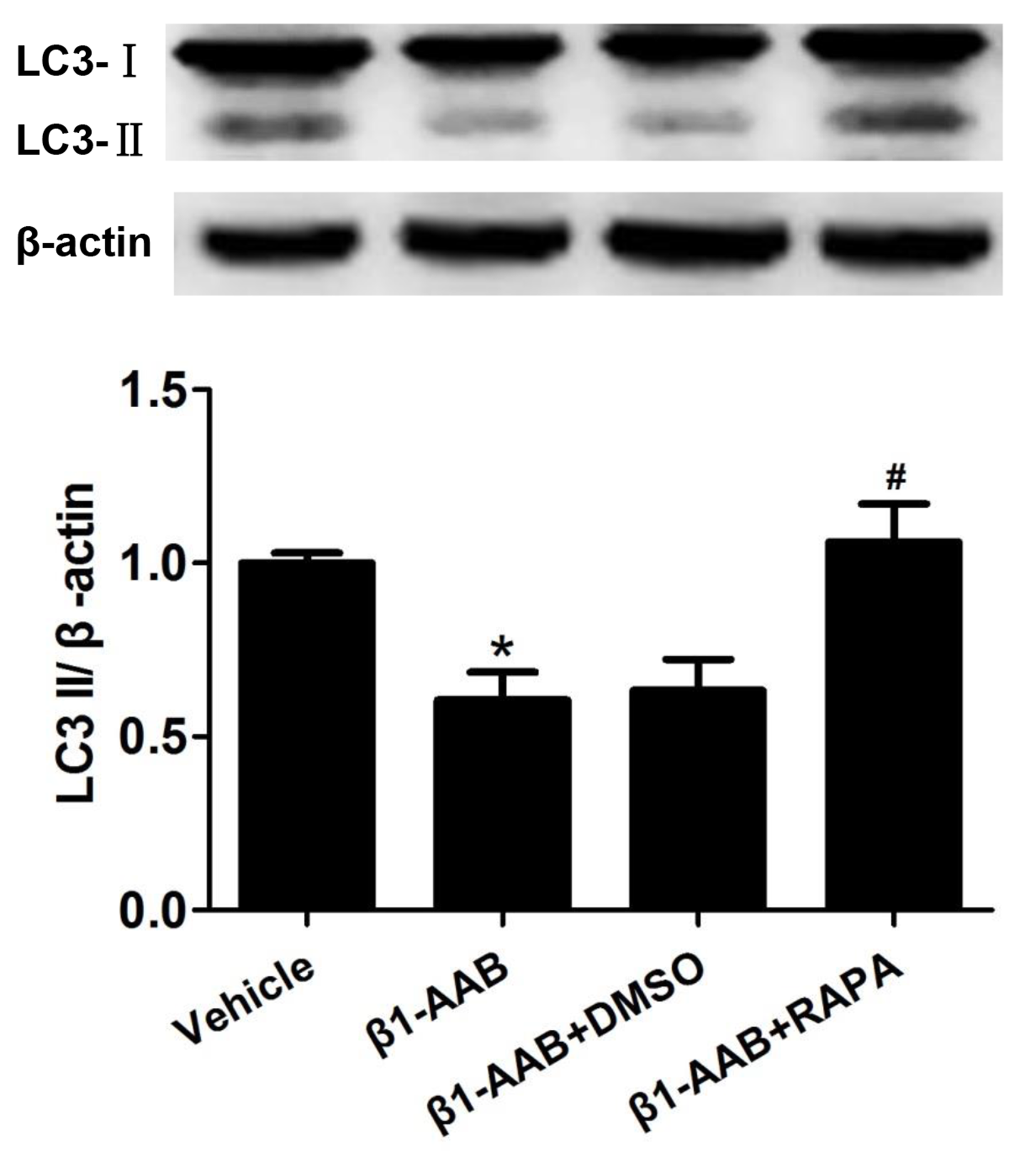

Supplement: Figure S4 — Pretreatment with RAPA increased the level of autophagy in β1-AAB-treated H9c2 cardiomyocytes. The differences in LC3 and Beclin-1 protein expression after treatment with RAPA. Data are expressed as Mean ± SD (n=6 per group). *P < 0.05 vs. Vehicle; #P < 0.05 vs. β1-AAB group. (TIF) [file pone.0081296.s004.tif]
