# Supplementary material for: Decreased Autophagy in Rat Heart Induced by Anti-β1-Adrenergic Receptor Autoantibodies Contributes to the Decline in Mitochondrial Membrane Potential
Source: PLoS One. 2013 Nov 20;8(11):e81296. doi: 10.1371/journal.pone.0081296 (PMC3835737; doi:10.1371/journal.pone.0081296)
Supplement: File S1 — Supplemental materials and methods. (DOC) [file pone.0081296.s005.doc]

**Supporting Information S1**

**Supplemental** **Materials and Methods**

**Cytotoxicity assay.**

H9c2 cells were plated in 96-well plates for 24h and subsequently treated with β_1_-AAB and β_1_-AR-EC_II_ seperately for 24h. Viable cells were determined using Cell Counting Kit-8 (CCK-8) assay. CCK-8 (Dojindo Molecular Technologies, CK04) was added (10 μl/well), and plates were incubated for a further 4h at 37 °C and then to measure the absorbance at 450 nm with a microplate reader using the following equation: viability % = [(AS - AB)/(AC - AB)] × 100%, where AS is the absorbance of the samples with β_1_-AAB or β_1_-AR-ECII, AC is the absorbance of the media of DMEM, and AB is the absorbance of the control.
